# Viruses utilize ubiquitination systems to escape TLR/RLR-mediated innate immunity

**DOI:** 10.3389/fimmu.2022.1065211

**Published:** 2022-11-25

**Authors:** Shanzhi Huang, Anchun Cheng, Mingshu Wang, Zhongqiong Yin, Juan Huang, Renyong Jia

**Affiliations:** ^1^ Avian Disease Research Center, College of Veterinary Medicine, Sichuan Agricultural University, Chengdu, China; ^2^ Institute of Preventive Veterinary Medicine, Sichuan Agricultural University, Chengdu, China; ^3^ Key Laboratory of Animal Disease and Human Health of Sichuan Province, College of Veterinary Medicine, Sichuan Agricultural University, Chengdu, China

**Keywords:** ubiquitination, virus, innate immune, E3 ubiquitin ligase, TLR, RLR

## Abstract

When the viruses invade the body, they will be recognized by the host pattern recognition receptors (PRRs) such as Toll like receptor (TLR) or retinoic acid-induced gene-I like receptor (RLR), thus causing the activation of downstream antiviral signals to resist the virus invasion. The cross action between ubiquitination and proteins in these signal cascades enhances the antiviral signal. On the contrary, more and more viruses have also been found to use the ubiquitination system to inhibit TLR/RLR mediated innate immunity. Therefore, this review summarizes how the ubiquitination system plays a regulatory role in TLR/RLR mediated innate immunity, and how viruses use the ubiquitination system to complete immune escape.

## Introduction

The ubiquitination system is extensively involved in the regulation of innate immune signaling during virus invasion. Pattern recognition receptors (PRRs), such as Toll like receptor (TLR) and retinoic acid-induced gene-I like receptor (RLR), will trigger various host countermeasures in response to pathogen-associated molecular patterns (PAMPs) or pathogen-induced cell physiology disturbance upon binding to ligand (1). In innate immune signaling, ubiquitin chains serve as platforms to facilitate protein-protein interactions that activate downstream innate immune pathways. Conversely, viruses will also utilize the host ubiquitination system to negatively regulate innate immune pathways and promote their proliferation. Therefore, the pivotal target for viruses to bypass antiviral signaling pathways is the ubiquitination system. Viruses utilize the ubiquitination system in ways including substrate molecular simulation, binding and blocking E3-substrate pairs, expressing virus-encoded E3s/deubiquitinating enzymes (DUBs), and hijacking host E3s/DUBs (2). Moreover, a method involving the packaging of ubiquitin chains into newborn virus particles for propagation in the host has recently been described (3).

**Figure 1:**
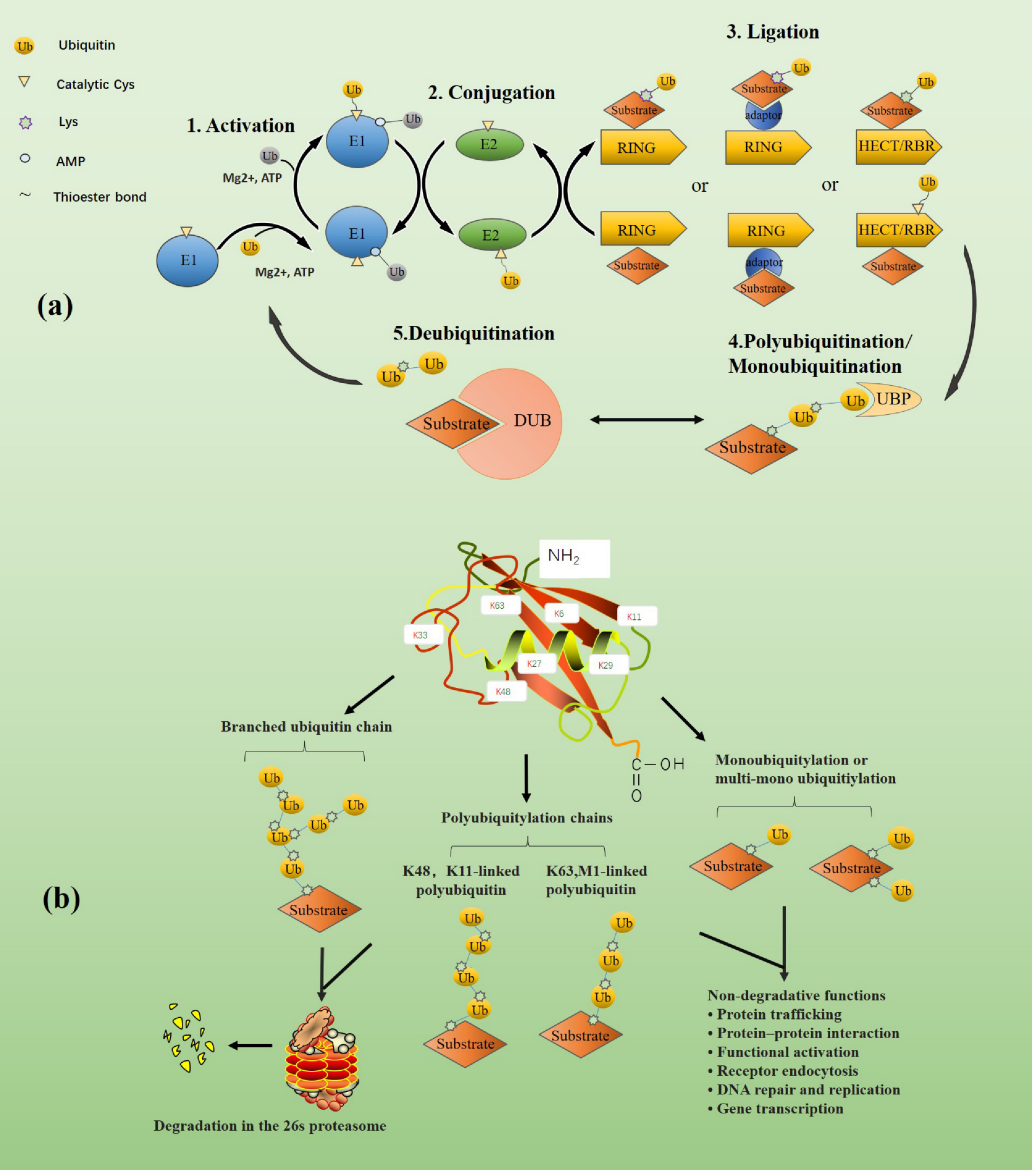
**(A)** The dynamic balance between ubiquitination and de-ubiquitination. First, in the ATP energy supply and the presence of magnesium ions, E1(blue) activates the ubiquitin by forming a covalent thioester bond through the cysteine at its catalytic site and the diglycine motif at the carboxyl end of ubiquitin (shown in yellow). The rest of ubiquitin (shown in gray) is non-covalently bound to the adenylation domain of E1 through its AMP-modified carboxyl terminal. The activated ubiquitin is transferred to the cysteine of the catalytic site of E2 through the diglycine motif of E1 to form the E2~ubiquitin thioester complex. Finally, E3 and E2-ubiquitin bind and jointly recognize and bind to the substrate. Depending on the type of E3, the way ubiquitin binds to the substrate is also different. For example, RING E3 ligase directly promotes the transfer of ubiquitin from E2 to the substrate. In contrast, HECT or RBR E3 ligase forms a thioester intermediate with ubiquitin before the transfer and then promotes the binding of ubiquitin to the substrate. Monoubiquitin chains or polyubiquitin chains are formed on substrates under the catalysis of ubiquitin-specific protease. Moreover, DUBs can deconjugate ubiquitin chains from ubiquitinated substrates, thereby reversing the ubiquitination process and regenerating free ubiquitin molecules. The figure is referenced from **Figure 1** of the ubiquitin system: a critical regulator of innate immunity and pathogen-host interactions (1). **(B)** The classification of ubiquitin chains and the functions of various ubiquitin chains. Different types of ubiquitination modifications lead to different physiological activities of substrates. Branched, K48-, and K11-linked ubiquitin chains typically target substrates for degradation in the 26s proteasome, while K63- and M1-linked ubiquitin chains and mono- or multi-mono-ubiquitin chains are mainly involved in non-proteolytic events, such as signaling cascades, protein interactions. The figure is referenced from **Figure 1** of Ubiquitin-binding domains - from structures to functions (7).

Ubiquitin (Ub) is a highly conserved protein found in most eukaryotic cells. Ubiquitin is composed of 76 amino acids with a molecular weight of around 8.6 kDa. Ubiquitination refers to the process of binding ubiquitin to specific target proteins under the synergistic effect of ubiquitin-activating enzyme E1, ubiquitin-conjugating enzyme E2, and ubiquitin ligase E3. The human genome encodes 2 E1s, about 40 E2s, and more than 600 E3s (4–6), of which E3s play a significant role in the specificity of ubiquitination. The specific process of ubiquitination is shown in the figure (**Figure 1A**). The isopeptide bond formed between the glycine residue at the carboxyl-terminal of ubiquitin and another ubiquitin amino-terminal methionine (M1) or the internal lysine (K) residue connects multiple ubiquitin to form polyubiquitin chains. The existence of seven Ks and M1 will create all sorts of ubiquitin chains, including K6-, K11-, K27-, K29-, K33-, K48-, K63-, and M1 connected homotype or multiple mixed ubiquitin chains. In addition, similar to the reversibility of phosphorylation, the conjugated ubiquitin chains can also be precisely cleaved by the DUBs, resulting in enhanced protein stability or weakened ubiquitination signal (1) (**Figure 1A**).

Ubiquitination was initially found to be involved in protein degradation, but later studies have found that ubiquitination can also mediate processes such as protein-protein interactions and cell signaling (8). For example, proteins modified by ubiquitin chains linked to K11 and K48 are typically targeted for degradation by the 26s proteasome (9, 10). When proteins are modified by M1 or K63-linked ubiquitin chains, these conjugates adopt extended conformations and enable reversible assembly of multiprotein complexes, which mainly involves in various non-proteolytic events. For example, K63-linked ubiquitination regulates kinase activation in the nuclear factor-κB (NF-κB) pathway (11). However, there are some exceptions, such as under autophagic conditions, the adaptor protein p62 is modified by K63-linked ubiquitin chains, which are recognized and degraded by lysosomes. Ubiquitination regulates thefunction, abundance, or subcellular distribution of proteins involved in almost every cellular process, and its role in modulating TLR/RLR mediated innate immunity is increasingly evident (**Figure 1B**).

In this review, we first introduce the role of the ubiquitination system in the TLR/RLR mediated innate immune pathway, further focus on how the body takes advantage of its ubiquitination system to negatively regulate the TLR/RLR mediated innate immune pathway and finally focus on how different viruses use the ubiquitination system to assist their proliferation.

## Ubiquitination is involved in TLR signaling

### Virus induced TLR signal transduction

At present, 13 TLR family members have been found in mammals, which recognize different PAMPs (12). TLR3, TLR7, TLR8, and TLR9 distributed in intracellular vesicles are primarily involved in recognizing virus-related ligands. For example, the double-stranded RNA (dsRNA) formed during virus replication is recognized by TLR3, the viral single-stranded RNA (ssRNA) is recognized by TLR7 and TLR8, and the unmethylated DNA sequence of the virus is recognized by TLR9. TLRs distributed on the cytoplasmic membrane are mainly involved in bacterial recognition. For example, TLR4 is mainly involved in the recognition of bacterial lipopolysaccharides (LPS).

**Figure 2:**
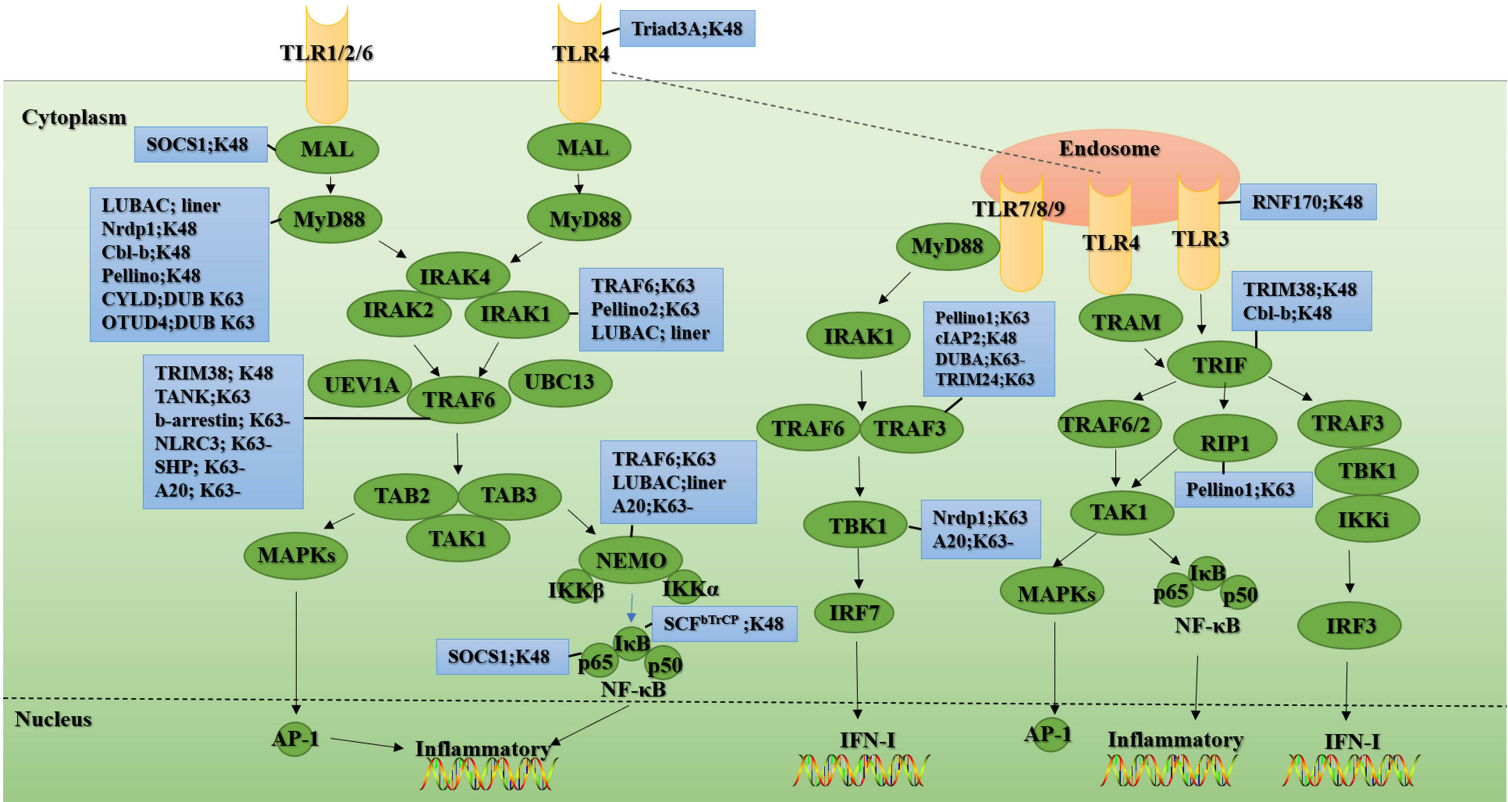
Ubiquitination and de-ubiquitination in the TLR signal. Upon ligand recognition, most TLR family members (shown in yellow), except TLR3, recruit the adaptor protein MyD88 and thus initiate a MyD88-dependent pathway involving the signal transduction mediators IRAKs, TRAF6, TAK1, TAB1, and TAB2, ultimately activating NF-κB transcription factors and MAPKs (shown in green), thereby inducing the production of IFN-I and inflammatory cytokines. Unlike other TLR family members, TLR3 (shown in yellow) signaling is completely dependent on TRIF (shown in green). This TRIF-dependent pathway mediates not only the production of proinflammatory cytokines but also the production of IFN-I upon activation of TLR3 and TLR4. In the blue boxes are the E3s or the DUBs involved in the regulation of this signaling protein. “-” means removing. The figure is referenced from **Figures 1** and **2** of Regulation of Toll-like receptor signaling in the innate immunity (18).

TLRs are classified into two categories based on whether they contain the signaling protein myeloid differentiation factor 88 (MyD88). Except for TLR3, all TLRs mediate immune signal transduction through MyD88-dependent pathways, while TLR3 induces cytokine production through MyD88-independent pathways (ie the Toll interleukin 1 receptor homology (TIR) domain-containing adapter-inducing interferon-β (TRIF) pathways) (13). Furthermore, TLR4 not only relies on MyD88 to complete signal transduction but also interacts with recombinant translocation-related membrane protein (TRAM) to target TRIF to complete downstream signal transduction (14). After TLR recognized the ligand, all TLRs except for TLR3 recruited the downstream signaling molecule MyD88 through their TIR domain. Upon ligand stimulation and the interaction of the death domains of the two molecules, MyD88 recruits IL-1 receptor-related kinase-4 (IRAK4). Due to conformational change, IRAK4-IRAK1 loses affinity for MyD88 and binds to tumor necrosis factor (TNF) receptor-associated factor 6 (TRAF6) through its TRAF6 binding motif to promote the activation of downstream signaling molecules. TRAF6 is an E3 that catalyzes the formation of K63-linked ubiquitin chains through Ubc13 and Uev1A to recruit and activate transforming growth factor-β (TGF-β) activated kinase 1 (TAK1), TAK1 binding protein 1 (TAB1), TAB2/3 kinase complex (15). Upon stimulation, TAB2 recognizes the unanchored K63-linked polyubiquitin chains to bind to TRAF6, thereby promoting the binding of TAK1 to TRAF6. This combination leads to the autophosphorylation of TAK1. Phosphorylated TAK1 will further activate the IκB kinase (IKK) complex and eventually lead to the activation of MAP kinase (c-Jun N-terminal kinase (JNK) -p38, MAPK) and NF-κB to induce the production of inflammatory factors (15–17) (**Figure 2**).

Unlike other members of the TLR family, TLR3 signaling is entirely dependent on the TRIF. This pathway can mediate the production of pro-inflammatory cytokines and type I interferon (IFN-I) (19–22) (**Figure 2**). TLR3 activates TRAF3 through TRIF, thereby recruiting IKK/TRAF family member-associated NF-κB activator (TANK) binding kinase 1 (TBK1) and promoting TBK1 phosphorylation. The phosphorylation of TBK1 further leads to the phosphorylation and nuclear translocation of IFN regulatory factor 3 (IRF3), thereby facilitating IFNβ expression. The TLR3-TRIF pathway also activates NF-κB in a similar way to the MyD88-dependent pathway and ultimately induces various inflammatory factors (**Figure 2**).

### Ubiquitination mediates TLR signaling

Activation of downstream signaling molecules NF-κB and IRF3 in TLR signaling requires polyubiquitination of intermediate signaling molecules such as MyD88, TRIF, TRAF6, TAB2/3, NF-κB essential modulator (NEMO), and TRAF3. Ubiquitination in TLR signaling is mainly divided into K48-linked and non-K48-linked ubiquitination. Generally, K48-linked ubiquitination is involved in the negative regulation of excessive innate immune response, while non-K48-linked ubiquitination promotes TLR signal transduction during pathogen infection.

MyD88 recruits signal intermediates including IRAK1, IRAK4, and TRAF6 through its death domain, and mediates NF-κB and IRF7 downstream signal transduction. TRAF6 further promotes IRF7 ubiquitination. Activated IRF7 undergoes nuclear translocation and drives the production of IFN-I (23). TRAF6 also encourages K63-linked ubiquitination of NEMO, which can recruit the TAB-TAK1 kinase complex (24). TRAF6 is a signal adaptor molecule essential for TLR signal transduction (25–27). TRAF6 binds to the Pro-Xaa-Glu motif at the carboxyl-terminal of IRAK1 and IRAK2 to trigger the dimerization of its really interesting gene (RING) domain and activate its E3 activity (28, 29). TRAF6 further cooperates with the E2 dimer Ubc13/Uev1A to catalyze the K63-linked ubiquitination of IRAK1 and promote IKK activation (16, 30). In addition, TRAF6 acts as an intermediate platform by binding to the K63-linked ubiquitin chain to assist in the recruitment and activation of TAK1 and IKK (31). NEMO and TAB1, and TAB2 have ubiquitin-binding functions. As mentioned earlier, TAB2/3 recruits TAK1 to ubiquitinated TRAF6.

However, the role of TRAF6 auto-ubiquitination in TLR signaling is controversial. Studies have shown that mutations in the key ubiquitination site K124 of TRAF6 severely impair the TRAF6-mediated NEMO ubiquitination, and the activation of TAK1 and IKK (32). Moreover, it was shown that although the RING finger (RF) domain of TRAF6 is necessary for the activation of TAK1, the auto-ubiquitination of TRAF6 is not necessary for the recruitment and activation of TAK1 (33). However, the above studies all emphasize the importance of the RING domain of TRAF6 for downstream signal transduction. It is generally believed that the key of MyD88 signal transduction is the E3 activity of TRAF6. However, when the E3 inactivated mutants of TRAF6 are expressed in TRAF6 ^-/-^ cells, these mutants can partially retain the TLR/IL-1 signal. It is worth noting that TLR/IL-1 signal is completely destroyed when the Pellino1 and Pellino2 inactivated mutants are expressed in TRAF6 ^-/-^ cells (34). In addition, impaired MyD88 signaling was found in knock-in mice of TRAF6 E3 activity deletion mutants. MyD88 signaling was also partially preserved in the primary macrophages and mouse embryonic fibroblasts (MEFs) of the mice (35). This indicates that Pellino1, Pellino2, and TRAF6 encourage the formation of MyD88-dependent K63-linked ubiquitin chains in certain cells, but the role of TRAF6 in this pathway has nothing to do with its E3 activity.

Similar to TRAF6, E3 Pellino can also be combined with E2 Ubc13-Uev1a to catalyze the formation of K63-linked polyubiquitin chains *in vitro*, but its activation way is different. Pellino1 realizes the conversion between active and inactive forms through reversible phosphorylation *in vitro*. Its phosphorylation and activation are mainly mediated by signal molecules such as IRAK1, IRAK4, IKK, and TBK1 (36–38). Furthermore, studies have shown that in IRAK-deficient cells, co-transfection of DNA encoding wild-type IRAK1 and Pellino2 can still facilitate the formation of the K63-linked polyubiquitin chains of IRAK and its binding to the NEMO complex, which indicates that the formation of K63-linked polyubiquitin chains can be assisted by Pellino subtypes, thereby contributing to the activation of downstream signals that depend on IRAK1/IRAK4 (36).

TLR3/4 agonists can cause the polyubiquitination of kinase receptor-interacting protein 1(RIP1), but unlike TRAF6, RIP1 itself does not have the function of E3. Therefore, there are speculations that TRAF6 may mediate the polyubiquitination of RIP1. Still, the absence of TRAF6 does not affect the signal transduction of the TRIF-dependent pathway, so the ubiquitination of RIP1 is mediated by another E3 (39, 40). A recent study found that the K63-linked polyubiquitination of RIP1 caused by TLR signal stimulation is mediated by Pellino1, a member of the highly homologous E3s family (41). Studies have shown that the loss of Pellino1 can disrupt the ubiquitination of RIP1 and lead to impaired NF-κB signaling upon TLR3/4 ligand poly(I:C) stimulation. Consistent with this, Pellino1^-/-^ mice are resistant to LPS-induced septic shock, so Pellino1 is essential for TRIF-dependent TLR signal transduction (41). In contrast, in the MyD88-dependent pathway, Pellino1 seems to have no activating effect on key intermediate signaling molecules but instead promotes MyD88 signaling by mediating the K48-linked ubiquitination degradation of TRAF3 (42). Recently, it has been discovered that during vesicular stomatitis virus (VSV) infection, the E3 Triple motif (TRIM) 24 catalyzes the binding of residues K429/K436 of TRAF3 to the ubiquitin chains linked to K63, so that TRAF3 can better bind to downstream signal molecules mitochondrial antiviral signal protein (MAVS; also known as VISA/Cardif/IPS-1) and TBK1, thereby promoting the production of related antiviral molecules (43).

The classical IKK complex is composed of three components: protein kinases IKKα and IKKβ, and NEMO (44, 45). Among them, NEMO, which has no catalytic activity, is pivotal for the activation of NF-κB signaling. The study found that NEMO contains a C-terminal zinc finger (ZF) domain and a new type of bipartite ubiquitin-binding domain (also known as NOAZ) that can recognize the K63-linked polyubiquitin chains (46, 47). Moreover, mutations in the above domains, such as NEMO[D311N], would prevent NEMO from undergoing K63-linked ubiquitination and impair IKK complex activation, leading to pigment incontinence and hypohidrosis ectoderm development poor and immunodeficiency. This indicates that the activation of the classical IKK complex requires NEMO, which is activated by ubiquitination. It was proposed that the IKK and TAK1 complex could be recruited through the K63-linked ubiquitin chains, thereby assisting TAK1-dependent IKK activation (48). The key to TAK1-mediated phosphorylation of IKKα and IKKβ is the linear ubiquitination of NEMO, which is mediated by the linear ubiquitin assembly complex (LUBAC) (49, 50). The activated IKKα continues to phosphorylate the IκBα subunit, causing it to be modified by the K48-linked ubiquitin chains and proteasome degradation, thereby releasing NF-κB from the cytoplasm and causing its nuclear translocation (49). With the deepening of research, it was discovered that NEMO can also bind to K48- and K27-linked ubiquitin chains (51). It will be very fascinating to explore the cross-regulatory effects of different types of polyubiquitination on NEMO.

LUBAC is a heterotrimeric complex composed of SHANK-associated RH domain-interacting protein (SHARPIN), heme-oxidized IRP2 ubiquitin Ligase 1 (HOIL-1), and HOIL-1 interacting protein (HOIP, catalytic subunit). LUBAC is also an E3, which mainly catalyzes the formation of linear ubiquitin chains (52–55). The discovery of LUBAC has taken a new twist in the regulation of the IKK complex. Studies have shown that the affinity of NEMO to the linear ubiquitin chains is one hundred times higher than that of the K63-linked ubiquitin chains (56, 57). Furthermore, in HOIL-1-deficient MEFs, IL-1-induced activation of the classical IKK complex is impaired (49), and the activation of the IKK complex is restored by adding LUBAC to the Hela extract lacking this E3 *in vitro* (58). Similarly, the IL-1-dependent linear ubiquitin chains could not be detected in the MEFs of the mutant knock-in mouse [C879S] in which the E3 HOIP was inactivated, and this blocked the activation of the classical IKK complex (59). From this, it can speculate that LUBAC-mediated linear ubiquitin chain production is indispensable for activating the classical IKK complex. A recent study showed that the activation of IKKβ was proved to be completed in two steps. First, TAK1 catalyzes the phosphorylation of IKKβ at Ser177, followed by IKKβ autophosphorylation at Ser181 (50). The IL-1-dependent phosphorylation of IKKα at Ser176 (or IKKβ at Ser177) was blocked in the MEFs of HOIP[C879S] or NEMO [D311N] mice. Therefore, the linear ubiquitination of NEMO is essential for TAK1-mediated activation of the IKK complex *in vivo* (50). The activation of IL-1-dependent P38α MAPK, JNK1, and JNK2 does not require the formation of linear ubiquitin chains (59).

### Ubiquitination negatively regulates TLR signaling

When compared to other types of ubiquitin chains, K48-linked polyubiquitin chains typically degrade the target protein *via* the proteasome and then quickly terminate signal transduction to prevent an excessive immune response from harming the body. In addition, the host DUBs can terminate innate immune signaling by removing the ubiquitin chain of the target protein. Such a negative regulation mechanism is abundant in TLR signals.

TLR itself is a target of ubiquitination degradation. For example, the RF protein Triad3A, as an E3, can mediate the degradation of TLR4/9 without affecting the expression and signal transduction of TLR2 (60). Similarly, RING finger protein (RNF) 170 is also an E3, which mediates K48-linked polyubiquitination after binding to TLR3 and leads to proteasomal degradation of TLR3 (61). Besides, under poly(I:C) stimulation, compared with mice lacking the RNF170 gene, the macrophages of wild-type mice initiate a weaker TLR3-mediated innate immune response, which further confirms the above conclusion (61).

Neuregulin receptor degradation protein-1 (Nrdp1) appears to play a dual role in TLR signaling. On the one hand, it mediates K48-linked ubiquitination and proteasomal degradation of the MyD88 to inhibit MyD88-dependent activation of NF-κB and activator protein-1 (AP-1), as well as the production of inflammatory factors. On the other hand, Nrdp1 mediates the K63-linked ubiquitination of TBK1, thereby promoting IFN-I production (62). Furthermore, the E3 casitas B-lineage lymphoma (c-Cbl) can also negatively regulate the TLR signaling by mediating the proteasome degradation of MyD88 and TRIF (63). Through biochemical purification and mass spectrometry analysis, atrophin-1 interacting protein 2 (AIP2) was identified as an interacting protein of TRIF. AIP2 interacts with TRIF and boosts its K48-linked polyubiquitination and subsequent proteasomal degradation. Moreover, the up-regulation of pro-inflammatory cytokines and IFN-I was detected in macrophages of AIP2 knockout mice (64). In addition, Pellino binds to MyD88 through its C-terminal extension domain and accumulates on the plasma membrane in a MyD88-dependent manner, promoting the ubiquitination and degradation of MyD88, thereby completing the negative regulation of TLR signaling (65). The K48-linked ubiquitination and proteasome degradation of MyD88 is also induced by TGF-β, and this process is mediated by the E3s Smad ubiquitination regulatory factor 1 (Smurf1) and Smurf2 recruited by Smad6, which ultimately negatively regulates the pro-inflammatory signal mediated by MyD88 (66).

Although many E3s are involved in catalyzing the ubiquitination of TRIF and MyD88, DUBs that mediate the de-ubiquitination of TRIF and MyD88 are rarely reported. Although infection with non-typeable *Haemophilus influenzae* has been reported to induce K63-linked polyubiquitin at the K231 residue of MyD88 to promote proinflammatory cytokine production, cylindromatosis (CYLD) will remove the K63-linked ubiquitin chain at this residue of MyD88 to negatively regulate MyD88-mediated signaling (67). Furthermore, studies have shown that DUB ovarian tumor family deubiquitinase 4 (OTUD4) negatively regulates TLR signaling by removing K63-linked ubiquitin chains on MyD88 (68). TRIM38 mediates the binding of TRAF6 and TRIF to the K48-linked ubiquitin chains to boost their proteasome degradation, thereby negatively regulating TLR signals and preventing excessive innate immunity from damaging the body (69, 70). The study found that in TANK^-/-^ cells, TLR-mediated activation of NF-κB was accompanied by increased polyubiquitination of TRAF6, so TANK may negatively regulate TLR signals by inhibiting the ubiquitination level of TRAF6 (71). Moreover, after TRAF6 ubiquitinates cellular inhibitor of apoptosis 2 (cIAP2), cIAP2 as an E3 will target the proteasome degradation of TRAF3, thereby restraining TLR4-mediated signal transduction (24, 72). β-arrestin belongs to the family of multi-functional proteins. It forms a complex with TRAF6 after TLR signal activation, and the formation of this complex inhibits the auto-ubiquitination of TRAF6, thereby inhibiting the activation of NF-κB and AP-1 (73). Similarly, nucleotide-binding leucine-rich repeat-containing receptor (NLR) family pyrin domain-containing 3 (NLRC3) can also interact with TRAF6 to attenuate K63-linked polyubiquitination on TRAF6, resulting in NF-κB signal transduction being blocked (74). As a DUB, DUBA can selectively weaken the K63-linked ubiquitination of TRAF3, assist in the dissociation of TRAF3 from the signal complex containing TBK1, and negatively regulate the production of IFN-I mediated by TRAF3 (75).

At rest, NF-κB binds to its inhibitor IκBα and is sequestered in the cytoplasm. Upon stimulation by upstream signals, IKKβ will specifically phosphorylate Ser32 and Ser36 at the N-terminus of IκBα. Phosphorylation at these sites is recognized by the SCFbTrCP complex. The SCFbTrCP complex will further promote K48-linked ubiquitination and proteasomal degradation of IkBa. Eventually, the p65 and p50 of NF-κB combine to form heterodimers, which are released from the cytoplasm and transferred to the nucleus, thereby promoting the production of downstream NF-κB-dependent genes (76–78).

Continuous incorrect activation of NF-κB signals will cause autoimmune diseases, cancer, and inflammation-related diseases. Therefore, the body will have a negative regulation mechanism of NF-κB signaling. The orphan nuclear receptor small heterodimer partner (SHP) has a dual role in regulating NF-κB signaling. Firstly, SHP prevents the trans-activation of p65 subunits. Secondly, SHP inhibits TRAF6 ubiquitination and the activation of NF-κB mediated by TLR signals (79). As a nuclear E3, PDZ-LIM domain-containing protein 2 (PDLIM2) can specifically bind to the p65 subunit of NF-κB to promote its nuclear dissociation and ubiquitin-dependent proteasomal degradation, thereby negatively regulating NF-κB signaling (80). Besides, mass spectrometry analysis revealed that suppressor of cytokine signaling 1 (SOCS1) is a binding ligand for p65. SOCS1 and p65 associate with each other only within the nucleus, SOCS1 further mediates K48-linked polyubiquitination and proteasomal degradation of p65 through its SOCS BOX, thereby limiting NF-κB signaling (81). SOCS1 also interacts with Mal (the adaptor protein for downstream signals of TLR2/4) and mediates its ubiquitination-dependent degradation to rapidly limit the innate immune response signal transduction (82).

The ubiquitin-modifying enzyme A20 is necessary for the termination of TLR signaling, which inhibits TLR signaling-induced proinflammatory factor production and NF-κB signaling by removing the polyubiquitin chains of TRAF6 (83), and it can also inhibit TRIF mediated NF-κB signaling to limit MyD88-independent TLR signaling (84). In addition, A20 can disrupt the binding of TBK1-IKKi to the K63-linked ubiquitin chains by acting independently of its de-ubiquitination domain, thereby limiting the activation of IRF3 downstream signals (85). In dendritic cells, the ubiquitin-binding association domain of rhomboid protease Rhbdd3 binds to the K27-linked polyubiquitin chains of NEMO and recruits A20. A20 further weakens the K63-linked polyubiquitination of NEMO and negatively regulates TLR-dependent NF-κB signaling (86).

## Ubiquitination is involved in RLR signaling

### Virus induced RLR signal transduction

Cytoplasmic virus dsRNA is recognized by RLR, including retinoic acid-inducible gene I (RIG-I), melanoma differentiation-associated protein 5 (MDA5), and laboratory of genetics and physiology (LGP2). Only RIG-I and MDA5 have an N-terminal caspase activation and recruitment domain (CARD) that can mediate downstream signaling. Although LGP2 does not have CARD, it regulates the activation of RIG-I and MDA5 (87). The C-terminal domain (CTD) of RIG-I and MDA5 is mainly involved in recognizing viral RNA. RIG and MDA5 recognize different types of viral RNA due to their different CTDs. For example, RIG-I identifies relatively short dsRNA (<1 kbp), while MDA5 identifies longer dsRNA (> 1 kbp) (88).

**Figure 3:**
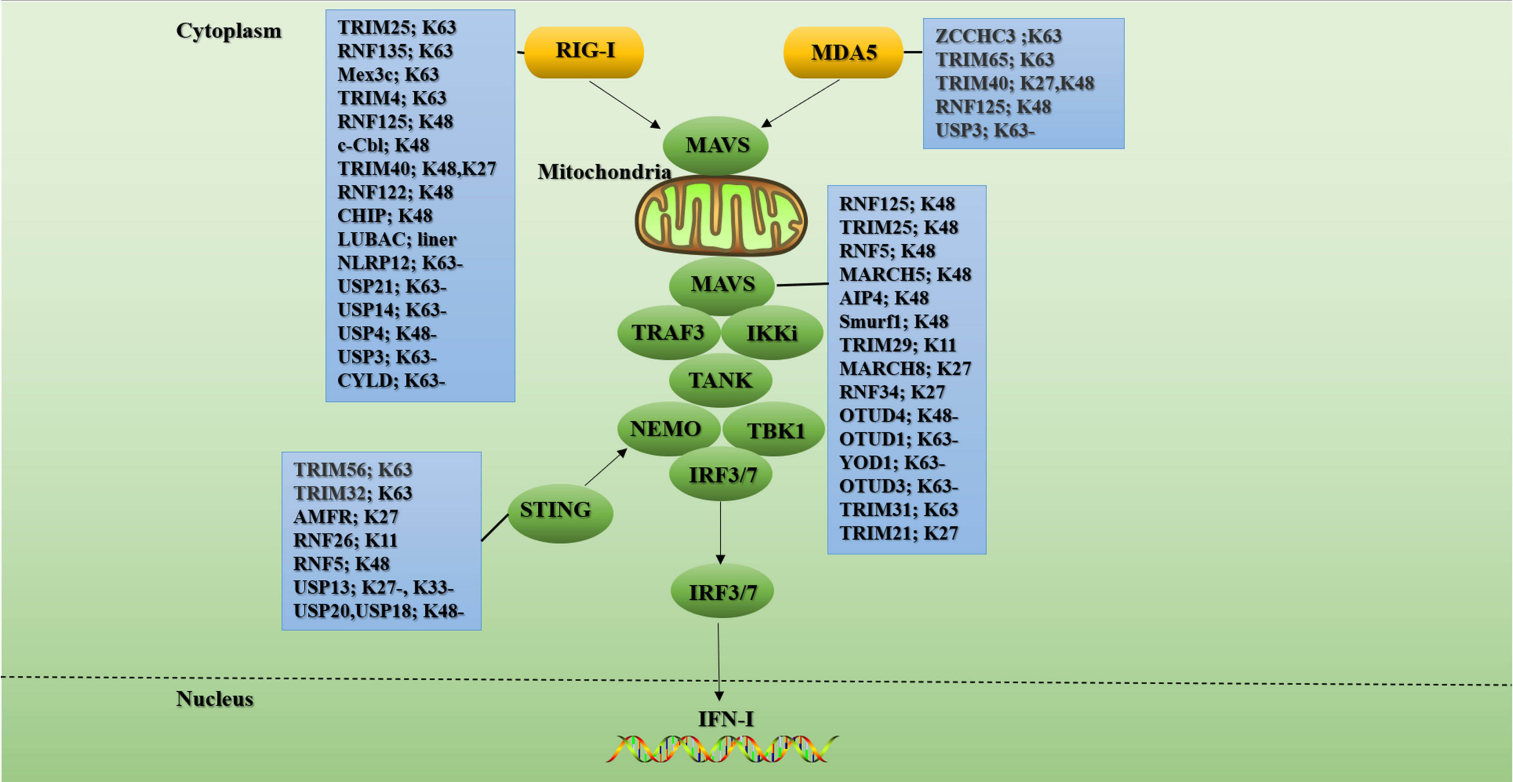
Ubiquitination and de-ubiquitination in the RLR signal. RIG-I/MDA5 undergoes significant conformational changes upon ligand binding. Then they bind to the adaptor protein MAVS on the outer membrane of mitochondrial through CARD-CARD homotypic interactions. These further triggers MAVS to recruit other signaling partners including TRAFs, TANK, and NEMO to activate IKKϵ and TBK1. Ultimately, the transcription factors IRF3 and IRF7 are phosphorylated by activated TBK1 and then translocated into the nucleus to initiate antiviral gene transcription. K63-linked polyubiquitination of signaling molecules such as RIG-I, MDA5, MAVs, and STING is critical for RLR signaling. Conversely, K48-linked ubiquitination and subsequent proteasomal degradation play important roles in fine-tuning RLR antiviral innate immunity. In the blue boxes are the E3s or the DUBs involved in the regulation of this signaling protein. “-” means removing. The figure is referenced from **Figure 2** of Ubiquitin in the activation and attenuation of innate antiviral immunity (2).

Virus infection activates RLR to promote the expression of two subtypes of IFN-I, IFN-α, and IFN-β, which stimulate the transcription of hundreds of IFN-stimulated genes (ISGs) (2). The IFN-I production pathway mediated by the RLR signaling pathway is as follows: As RIG-I and MDA5 recognize PAMPs together, proteins assemble along dsRNA to form nucleoprotein filaments (89, 90). The formation of nucleoprotein filaments leads to the binding of RIG-I-CARD molecules to produce a 2CARD tetramer structure (91). This tetramer structure, as the core of MAVS oligomerization, leads to the formation of MAVS prion-like fibers, which are activated as an important intermediate in the downstream signaling pathway (92). MAVS coordinates and recruits TRAFs, TANK, NEMO, and other proteins to co-localize on the outer membrane surface of mitochondria, thereby activating TBK1 and IKKϵ. TBK1 and IKKϵ are responsible for the activation of IRF3, 7, and NF-κB, respectively. The activated IRF-3, 7, and NF-κB enter the nucleus and cooperate to initiate IFN-I and inflammatory cytokines expression (93) (**Figure 3**). MAVS is a crucial signal platform that links mitochondria with intracellular antiviral signals. Furthermore, the endoplasmic reticulum (ER) is also an intermediate that mediates signal transduction, and the ER protein stimulator of IFN genes (STING) plays a vital role in this process (94–96) (**Figure 3**).

### Ubiquitination of RIG-I and MAD5

RIG-I has many complex post-translational modifications (PTMs), among which polyubiquitination modification is one of the most important modifications for RIG-I activation and degradation. It is worth noting that although MDA5 protein is mainly regulated by phosphorylation, there are also reports on polyubiquitination modification of MDA5 protein in recent years. For example, the K174 residue of MDA5 CARD activates the downstream pathway by binding to the K63-linked polyubiquitin chain (97). In addition, the K174A ubiquitination-deficient mutant of MDA5 failed to induce IFN-I production (98). Current studies have found that TRIM25 and TRIM65 mediate the K63-linked ubiquitination of MDA5. The RNA co-sensor zinc finger CCHC-type containing 3 (ZCCHC3) promotes the K63-linked ubiquitination of MDA5 by recruiting TRIM25 (99), while TRIM65 directly catalyzes the K63-linked ubiquitination of the K743 residue in the MDA5 helicase domain (100). Furthermore, TRIM40 binds to the first CARD of MDA5 to promote K27- and K48-linked polyubiquitination and subsequent proteasomal degradation of MDA5 (101). However, how these E3 ligases cooperate to regulate MDA5-mediated innate immune responses needs to be further explored.

RIG-I mainly undergoes modification of ubiquitin chains linked by K48 and K63. These two types of ubiquitin modifications play a pivotal role in the activation and regulation of the RIG-I signaling pathway. Currently, four E3s have been reported to assist in K63-linked ubiquitination of RIG-I: TRIM25, RNF135 (also known as Riplet/REUL), Mex-3 RNA-Binding Family Member C (Mex3c), and TRIM4. Studies have shown that MEX3c, TRIM25, and TRIM4 can synergistically catalyze the ubiquitination of multiple lysine residues in RIG-I CARD domain to promote downstream signal transduction. Unlike E3 above, RNF135 appears to play an associated role primarily in the CTD domain of RIG-I.

Many studies have proved the critical role of TRIM25 in the activation of the RIG-I signaling. Gack et al. reported for the first time that TRIM25 mediated the K63-linked ubiquitination at the K172 residue of the N-terminal CARD of RIG-I, which was essential for downstream immune signal activation (102). Further studies showed that TRIM25 could not bind to RIG-I after the mutation of threonine 55 residue in the first CARD of RIG-I. Furthermore, after the mutation of K172 residue in the second CARD of RIG-I, the related ubiquitination level was down-regulated, indicating that TRIM25 first binds to the first CARD of RIG-I and then mediates the ubiquitination of the second CARD of RIG-I (103).

K63-linked polyubiquitin modification can stabilize the RIG-I 2CARD tetramer and drive the mitochondrial accumulation of RIG-I, assist the binding between RIG-I and MAVS, and activate MAVS to target mitochondrial signals to stimulate downstream signaling pathways. But it seems that as a means to limit the selection of escape mutations, in addition to the above activation mechanism, more ways have been found to activate RIG-I. For example, TRIM4 can not only act on K172 of RIG-I, but it can also mediate the K63-linked ubiquitination at residues K164 and K154, thereby participating in the activation of RIG-I (104). Mex3c-mediated RIG-I ubiquitination is attenuated due to amino acid substitutions at K45, K99, and K169 (105). However, contrary to previous studies, the activation of MAVS after RIG-I stimulation will not be interrupted by the knockout of TRIM4 and Mex3c (106).

Studies have shown that the deficiency of E3 RNF135 can cause severe damage to the innate antiviral immune response in mice, such as increased mortality and blocked induction of IFN-I in mice infected with the virus (107). RNF135 can bind to K909, K907, K888, K851, K849, and K788 of RIG-I CTD and mediate its K63-linked ubiquitination (108, 109). Furthermore, RNF135 also promotes ubiquitination of RIG-I at multiple sites, including 2CARD, and generates unanchored polyubiquitin chains that activate RIG-I signaling (110, 111). Moreover, studies have shown that RNF135 can facilitate the combination of TRIM25 and RIG-I CARD (109). The specific mechanism is: RNF135 triggers the open conformation of RIG-I and exposes RIG-I CARD by mediating the combination of K788 residues in the CTD of RIG-I with the K63-linked ubiquitin chains. Then TRIM25 catalyzes the ubiquitination of RIG-I CARD, which eventually promotes the interaction of RIG-I with MAVS and TBK1 (109). Supporting the above conclusion is that RNF135 knockdown can inhibit the association between RIG-I and TRIM25 and eliminate the recruitment of TBK1 (108). However, the role of RNF135 in the RIG-I signaling pathway remains controversial. Studies have shown that RNF135 can activate IRF3 in a RIG-I-independent way (112). Furthermore, it was found that RNF135 can synergistically boost the downstream RIG-I signaling in a ubiquitination-dependent and independent manner. On the one hand, RNF135 recognizes and ubiquitinates RIG-I pre-oligomerized on dsRNA. On the other hand, on longer dsRNA, RNF135 can bridge RIG-I filaments and induce aggregate-like RIG-I assembly (111). Why does RIG-I require four E3s to participate in its ubiquitination modification, and do these different E3s have redundant functions, or do they act in an environment- or cell-type-dependent manner? This is worthy of our further exploration.

Currently, there are mainly these E3s that mediate the K48-linked ubiquitination of RIG-I: RNF125, c-Cbl, TRIM40, RNF122, and CHIP (also known as STIP1 homology and U-box-containing protein 1(STUB1)). IFN-α and the dsRNA analogue poly(I:C) induce the production of RNF125, and RNF125 promotes the K48-linked polyubiquitin chains labeling of RIG-I, MDA5, and MAVS, and their proteasomal degradation, ultimately inhibiting the production of IFN-I (113). RNA virus infection upregulates Siglec-G, a member of the lectin family, through RIG-I dependent mechanism. The upregulated Siglec-G induces SHP2 and E3 c-Cbl to recruit to RIG-I, which eventually leads to c-Cbl assisted in K48-linked ubiquitination of RIG-I at K813 residue and proteasome degradation of RIG-I, thus limiting the production of IFN-I (114). TRIM40 not only mediates the degradation of MDA5 but also promotes the binding of RIG-I to K27 and K48-linked ubiquitin chains, leading to its proteasomal degradation. Knockout of TRIM40 has been shown to significantly enhance RLR signaling and antiviral immune response in mice (101). RNF122 is an E3 that is significantly expressed in macrophages. Wang et al. found that in RNA virus-infected cells, RNF122 can directly bind to RIG-I CARD, and RNF122 promotes K48-linked ubiquitination of K115 and K146 residues of RIG-I CARD through its RF domain, resulting in proteasome degradation of RIG-I and limiting RIG-I mediated antivirus signal (115). Mixed lineage leukemia 5 (MLL5) is an essential epigenetic modifier distributed in both cytoplasm and nucleus. Studies have shown that upon virus infection, MLL5 is translocated from the nucleus to the cytoplasm, and part of MLL5 interacts with RIG-I and E3 CHIP to boost CHIP to catalyze the K48-linked ubiquitination of RIG-I, thereby inhibiting innate immunity (116). Since the over-activation of RLR signals can lead to auto-immune diseases, the above-mentioned E3s that mediate RLR K48-linked ubiquitination may play a key role in RLR negative feedback. Still, the clinical application of these E3s in innate immunity remains to be explored.

In addition to negatively regulating RLR signaling by mediating K48-linked ubiquitination and proteasome degradation of RLR receptors, hosts can also negatively regulate RLR signaling through LUBAC and DUBs. A recent study showed that LUBAC suppressed virus-induced IFN-I by targeting the RIG-I/TRIM25 signal axis. The specific mechanism is: Firstly, HOIL-1L and HOIP boost the connection of K48-linked ubiquitin chains to TRIM25 to mediate its proteasomal degradation. Secondly, HOIL-1L competes with TRIM25 to bind to RIG-I, so these two mechanisms effectively inhibit the K63-linked ubiquitination of RIG-I and the production of IFN-I (117). In addition, the NLR family pyrin domain-containing 12 (NLRP12) can physically bind TRIM25 to reduce TRIM25-mediated K63-linked ubiquitination of RIG-I (118). Recently, four DUBs Ub specific protease 21 (USP21), USP14, USP3, and CYLD mediate negative regulation of RIG-I ubiquitination. Both USP21 and CYLD can remove the K63-linked ubiquitination of RIG-I and restrict RIG-I-mediated innate immunity (119, 120). USP3 can simultaneously encourage the de-ubiquitination of RIG-I and MDA5 (121), and USP14 catalyzes the de-ubiquitination of RIG-I through its USP domain (122). Unlike the DUBs mentioned above, USP4 is the only DUB that removes the K48-linked polyubiquitin chains from RIG-I to increase the stability of RIG-I (123).

### Ubiquitination of MAVS

MAVS plays a connecting role in RLR signal transduction, so its ubiquitination modification plays a vital important role in RLR signal transduction. Most of the ubiquitination of MAVS only occurs during viral infection. At present, at least 6 E3s lead to the ubiquitination mediated degradation of MAVS. For example, RNF125 furthers the ubiquitination and proteasome degradation of MAVS (113). TRIM25 mediates K48-linked ubiquitination of K7 and K10 residues of MAVS, leading to its proteasomal degradation and inhibition of IFN-I production (124). The study found that the expression of E3 MARCH5 is up-regulated during virus infection, and it can catalyze the ubiquitination of K7, and K500 residues of MAVS to mediate the proteasomal degradation of MAVS (125). Furthermore, E3 RNF5 also interacts with MAVS during viral infection, and it catalyzes K48-linked ubiquitination of K362 and K461 residues of MAVS, leading to its degradation by the proteasome (126). Poly(C)-binding protein 2 (PCBP2) is also one of the negative regulators of MAVS-mediated signaling. During viral infection, PCBP2 is induced to recruit the HECT domain-containing E3 ligase AIP4 by interacting with MAVS. AIP4 further promotes the K48-linked ubiquitination of K371 and K420 residues of MAVS, which eventually leads to the proteasomal degradation of MAVS (127). Besides, Nedd4 family interacting protein 1(Ndfip1) is also involved in the negative regulation of MAVS-mediated antiviral signaling. Ndfip1 furthers the polyubiquitination and degradation of unknown sites of MAVS by recruiting the E3 Smurf1 (128). Unlike the above-mentioned E3, TRIM29 mediates its degradation by catalyzing the binding of the polyubiquitin chains linked by K11 instead of K48 to MAVS (129). Furthermore, MARCH8 and RNF34 degrade MAVS in a unique way by catalyzing the ubiquitination of MAVS for autophagy (130, 131). It is worth noting that MARCH 8 interacts with MAVS in a CD317-dependent manner. Meanwhile, RNF34 directly binds to MAVS and promotes the binding of polyubiquitin chains linked by K27 and K29 to K297, K362, K348, and K311 residues of MAVS, and this ubiquitination modification is a key signal for inducing nuclear dot protein 52 kDa-dependent autophagic degradation.

Up to now, only four DUBs, OTUD4, OTUD3, OTUD1, and YOD1 have been found to mediate the de-ubiquitination of MAVS. Among them, OTUD4 is induced in an IRF3/7-dependent manner, and OTUD1 increases the stability of MAVS during viral infection by directly removing the K48-linked ubiquitination of MAVS, thereby promoting RLR-mediated innate immune signals (132). During RNA virus infection, OTUD1 is induced through the NF-κB pathway, and OTUD1 deubiquitinates Smurf1 to up-regulate the protein level of Smurf1 in cells. Smurf1 directly binds to signal components such as MAVS, TRAF3, and TRAF6 and catalyzes their ubiquitination and proteasome degradation to negatively regulate the production of IFN-I (133). As a member of the ovarian tumor family, YOD1 is recruited to mitochondria following viral infection. YOD1 interacts with MAVS through its ubiquitin regulatory X and ZF domains and removes K63-linked polyubiquitin chains on MAVS, thereby inhibiting the formation of MAVS prion-like aggregates (134). OTUD3 is an acetylation-dependent DUB. Studies have found that OTUD3 can directly bind to MAVS and remove its K63-linked ubiquitin chains to turn off the antiviral signal (135).

K63-linked ubiquitination of MAVS plays a crucial role in the recruitment of IKKϵ, IRF3, and the occurrence of antiviral signaling. A recent study found that viral infection leads to the recruitment of TRIM31 to mitochondria. TRIM31 further catalyzes the K63-linked polyubiquitination of K461, K311, and K10 residues of MAVS, which ultimately promotes the accumulation of MAVS and antiviral signal transduction (136). Furthermore, viral infection induces TRIM21 production in an IFN/Janus kinase/signal transducer and activator of transcription-dependent pathway, and loss of TRIM21 results in impaired innate immune responses. The specific mechanism is that TRIM21 interacts with MAVS through its PRY-SPRY domain, and TRIM21 catalyzes the binding of the K325 residue of MAVS to the K27-linked polyubiquitin chains through its RING domain, thereby promoting the innate immune response (137). This is consistent with the observation of interactions that TBK1 undergoes K27- but not K63-linked ubiquitination *in vitro* (138). However, the reason why so many E3s are involved in the regulation of MAVS is still unknown. Therefore, the research on solving the dynamic network relationship of polyubiquitination connected by K48 and K63 on MAVS will be very meaningful.

### Ubiquitination of STING

The ubiquitination of STING is also a crucial part of immune signal transduction. For example, TRIM56 and TRIM32, these two E3s can interact with STING to facilitate the binding of K63-linked ubiquitin chains to STING, thereby facilitating the production of IFN-I (139, 140). STING recruits autocrine motor factor receptors (AMFRs) upon stimulation of cytoplasmic DNA. AMFR interacts with STING in an insulin-inducible gene 1-dependent manner and catalyzes the binding of K27-linked polyubiquitin chains to STING, thereby recruiting TBK1 to generate antiviral genes (138). RNF26 promotes K11-linked ubiquitination of STING, and it can effectively prevent STING from RNF5-mediated K48-linked polyubiquitination and proteasome degradation. This is because they compete to catalyze the polyubiquitination of the K150 residue of STING (141, 142). It was found that DUB USP18 can recruit USP20 to remove K48-linked polyubiquitin chains on STING and enhance the stability of STING, which will benefit the production of downstream IFN-I and antiviral signals (143).

The ubiquitination of RLR signaling is a very complex process, and the same protein may involve multiple E3s or DUBs to regulate its ubiquitination. However, what is the inextricable relationship between them, and whether these studies can be used to provide possibilities for the design of immune drugs remains to be considered.

## Viruses use ubiquitination system to escape innate immunity

Because ubiquitination plays a vital regulatory role in innate immunity, it is not surprising that viruses take advantage of this feature of ubiquitination to promote their proliferation in the host. Viruses mainly manipulate the ubiquitination system through substrate molecular simulation, binding and blocking E3-substrate pairs, expressing virus-encoded E3s/DUBs, and hijacking host E3s/DUBs. In the following, we will summarize the relevant research in recent years.

### Substrate molecular simulation

There is no shortage of such examples in recent research. For example, recent research indicates that the rotavirus non-structural protein (NSP1) can target the proteasome degradation of β-transducin repeat-containing protein (β-TrCP). β-TrCP is a recognition substrate for the multi-subunit E3 complex Skp1/Cul1/F-box, which catalyzes the degradation of IκBα by recognizing β-TrCP to promote NF-κB signaling activation. Therefore, the degradation of β-TrCP will assist the virus to escape innate immunity (144). Another example is the varicella-zoster virus immediate-early protein open reading frame 61 (ORF61) interacts with phosphorylated IRF3. It mediates the ubiquitination of IRF3 and subsequent proteasome degradation to antagonize the production of IFN-β (145).

Proteomics analysis revealed that the human cytomegalovirus (HCMV) protein pUL21a interacts with the anaphase-promoting complex (APC). APC is an E3, which has a certain limiting effect on virus infection by regulating the ubiquitination and degradation of cell cycle regulatory proteins, while pUL21a mediates the ubiquitination and proteasome degradation of APC and destroys the restriction of APC to viral infection and facilitates HCMV infection (146). HCMV also utilizes its own encoded US7/US8 proteins to inhibit signaling downstream of TLR3/TLR4. The specific mechanism is as follows: Firstly, US7 interacts with ER-related degradation components Derlin-1 and Sec61 to promote TLR3/4 ubiquitination and proteasomal degradation. Secondly, US8 mediates their degradation by targeting TLR3/TLR4 to the proteasomal or lysosomal pathways (147). It was found that the hemagglutinin (HA) protein of IAV can further the ubiquitination and degradation of IFNAR, thereby destroying the host’s innate immune system (148) (**Table 1**).

**Table 1:** Substrate molecular simulation.

Virus protein	Target	Effect
Rotavirus NSP1	β-TrCP	NF-κB signal ↓
Varicella-zoster virus ORF61	IRF3	IFN-β production ↓
HCMV pUL21a	APC	Viral infection ↑
HCMV US7/US8	TLR3/TLR4	Viral infection ↑
IAV HA	IFNAR	Viral infection ↑

↑ increases; ↓ decreases.

### Binds and blocks E3-substrate pairs

It is not uncommon for viruses to antagonize innate immunity by blocking or binding substrate pairs of the ubiquitin system. Studies have found that during RNA virus infection, Trithorax group protein MLL5 will be transported from the nucleus to the cytoplasm to further the binding of E3 STUB1 and RIG-I. STUB1 catalyzes the K48-linked ubiquitination of RIG-I and mediates its proteasome degradation, thereby inhibiting the antiviral immune response (116). Viruses can also promote this process by using their own encoded protein. For example, Porcine delta coronavirus (PDCoV) nucleocapsid (N) protein can suppress the expression of IFN-I induced by VSV infection or poly(I:C) stimulation. The specific mechanism is: pRiplet is an E3, which can catalyze the binding of RIG-I and K63-linked ubiquitin chains to activate the RLR signal and boost the production of IFN-I, while the PDCoV N protein interacts with pRiplet, thereby shutting down the ubiquitination process and inhibiting the innate antiviral response (149). Besides, through mass spectrometry analysis, it was found that PDCoV N protein could also interact with IRF7 and facilitate its ubiquitination-mediated proteasome degradation, thereby restricting the expression of IFN-I and promoting virus proliferation (150).

The non-structural proteins of the viruses seem to play a vital role in this link. IAV NS1 protein inhibits antiviral response by inhibiting TRIM25-mediated RIG-I ubiquitination (151). Further studies have found that the binding of NS1 will affect the positioning of the CTD PRY/SPRY of TRIM25, which in turn will affect the binding of the ubiquitin chains linked by K63 to RIG-I (152). Moreover, NS1 of avian and human IAV strains bind to TRIM25 in a species-specific manner. It is worth noting that some IAV strains will also attach to another E3 RNF135, and this E3 will also catalyze the binding of RIG-I to the K63-linked ubiquitin chains (153). Similarly, Respiratory syncytial virus NS1 binds to the PRY/SPRY domain of TRIM25, thereby blocking the K63-linked ubiquitination of RIG-I CARD (154). West Nile virus NS1 protein binds to RIG-I instead of TRIM25 to form steric hindrance and affect the ubiquitination of RIG-I (155). Hepatitis C virus (HCV) NS3/4A is a serine protease that limits the expression of IFN-I. Studies have found that when NS3/4A is overexpressed *in vivo*, RNF135-mediated K63-linked ubiquitination of the RIG-I CTD domain is blocked. Meanwhile, this blocking is accompanied by a weakening of the combination between TRIM25 and RIG-I. Therefore, HCV antagonizes the innate immune pathway by targeting RNF135 and affecting the K63-linked ubiquitination of RIG-I (109, 156). Porcine reproductive and respiratory syndrome virus encoded nsp1α can interact with HOIP/HOIL-1L to weaken the combination of HOIP and SHARPIN, which not only affects the formation of the LUBAC complex but also weakens the linear ubiquitination of NEMO that depends on LUBAC, which ultimately leads to impaired NF-κB signaling (157).

TRIM25 and TRIM6 seem to be vital targets for viral proteins to suppress innate immunity. Paramyxovirus V protein is a known inhibitor of RLR signaling. Research indicates that V protein blocks the ubiquitination of RIG-I catalyzed by TRIM25 by combining with RIG-I and TRIM25, thereby destroying RLR signaling (158). In addition, the oncoprotein E6 encoded by human papillomavirus (HPV) targets the TRIM25-USP15-RIG-I axis. Normally, USP15 deubiquitinates TRIM25 to maintain TRIM25 stability, enabling TRIM25 to effectively promote RIG-I ubiquitination and activation. However, HPV E6 protein combines with TRIM25 and USP15 to boost the ubiquitination and degradation of TRIM25, which ultimately disrupts RIG-I signal transduction (159). Studies have found that the nucleocapsid protein of severe acute respiratory syndrome coronavirus (SARS-CoV) can also bind to the SPRY domain of TRIM25, which affects the binding between TRIM25 and RIG-I and disrupts TRIM25-mediated ubiquitination activation of RIG-I, eventually leading to impaired signal transduction downstream of RIG-I (160). Viral RNA can also bind to TRIM25 to inhibit RIG-I signaling. Sub-genomic flavivirus RNA (sfRNA) is a unique non-coding RNA of flavivirus, which is the incomplete degradation product of cellular 5’-3’ exoribonuclease 1 (XRN1) (161–163). It was found that the sfRNA of Dengue virus (DENV) serotype two strain (PR-2B strain) binds TRIM25 to block TRIM25 de-ubiquitination and attenuate RIG-I signaling, finally achieving the unique immune escape mechanism of Flaviviridae virus (164).

Nipah virus (NiV) is a highly pathogenic virus belonging to the Paramyxoviridae family. Studies have shown that NiV matrix protein binds to TRIM6 and degrades TRIM6, leading to the reduction of unanchored K48-linked ubiquitin chains of IKKϵ catalyzed by TRIM6, which ultimately disrupts the oligomerization and phosphorylation of IKKϵ and weakens production of IFN-I (165). Furthermore, Ebola virus (EBOV) VP35 binds to polyubiquitin chains non-covalently. Meanwhile, VP35 also binds to TRIM6, antagonizes IFN-I production by ubiquitinating TRIM6, and ultimately promotes EBOV replication (166) (**Table 2**).

**Table 2:** Binds and blocks E3-substrate pairs.

Virus protein/RNA	Target	Effect
PDCoV N	pRiplet IRF7	RLR signal ↓ IFN-I production ↓
IAV NS1	TRIM25 RNF135	RLR signal ↓ RLR signal ↓
RSV NS1	TRIM25	RLR signal ↓
WNV NS1	RIG-I	RLR signal ↓
HCV NS3/4A	RNF135	IFN-I production ↓
PRRSV nsp1α	HOIP/HOIL-1L	NF-κB signal ↓
Paramyxovirus V	RIG-I and TRIM25	RLR signal ↓
HPV E6	TRIM25 and USP15	RLR signal ↓
SARS-CoV	TRIM25	RLR signal ↓
DENV sfRNA	TRIM25	RLR signal ↓
NiV-M	TRIM6	phosphorylation of IKKε ↓, IFN-I production ↓
EBOV VP35	TRIM6	IFN-I production ↓

↑ increases; ↓ decreases.

### Express virus-encoded E3s/DUBs

Virus-encoded E3s typically degrade key signaling molecules in innate immunity to evade immune responses, while virus-encoded DUBs generally have a papain-like protease (PLP) structure that targets relevant immune factors. Studies have found that the arterial virus family has a PLP2 conserved domain with DUB activity, which can inhibit RIG-I ubiquitination, antagonize RLR innate immune signals, and facilitate the virus to escape innate immunity (167). Similarly, the OTU domain-containing-like protease encoded by the Nairo virus has DUB activity, and this structure also inhibits RIG-I ubiquitination. Furthermore, the catalytic domain PLP2 of mouse hepatitis virus A59 NSP3 can not only remove the ubiquitin chains of TBK1 and reduce its kinase activity but also de-ubiquitinate IRF3 and prevent IRF3 nuclear translocation, thus inhibiting the production of IFN-I (168, 169). Similarly, the precursor protease (L(pro)) of the foot-and-mouth disease virus (FMDV) is also a kind of PLP2. Further research found that L (pro) can significantly inhibit the ubiquitination of RIG-I, TRAF3, TRAF6, and TBK1 to inhibit the production of IFN-I, thereby promoting FMDV proliferation (170). Human coronavirus and SARS-CoV PLP can also reduce the ubiquitination level of proteins such as STING, RIG-I, TBK1, and IRF-3, thereby interfering with the production of IFN-I (171). Porcine epidemic diarrhea virus (PEDV) also encodes PLP2, which antagonizes IFN-I production by deubiquitinating RIG-I and STING (172). Moreover, the papain-like cysteine protease domain (PCP) encoded by the hepatitis E virus (HEV) ORF1 also has DUB activity, which antagonizes RLR signaling by deubiquitinating RIG-I and TBK1 (173).

There is also a class of virus-encoded proteins that directly have DUB activity. It was found that HSV VP1-2 can remove the ubiquitin chains of STING and block the phosphorylation activation of downstream genes TBK1 and IRF3, to reduce the production of IFN-I and assist HSV in immune escape in the brain (174). Besides, the Ubiquitin-specific protease UL36 (UL36USP) encoded by HSV is also a DUB. Firstly, UL36USP can block IFN-stimulated DNA-induced IFN-β production and NF-κB activation. Secondly, UL36USP can de-ubiquitinate IκBα to prevent its degradation, which blocks the activation of NF-κB (175). As a DUB, Hepatitis B virus (HBV) X protein (HBx) can remove the K63-linked polyubiquitin chains on RIG-I and TRAF3, resulting in the obstruction of downstream signal transduction (176). Seneca Valley Virus (SVV) 3C protease (3Cpro) also has DUB activity. Studies have found that the Cys160 and His40 residues of 3Cpro are essential sites for its de-ubiquitination activity. 3Cpro widely promotes RIG-I, TBK1, and TRAF3 de-ubiquitination to stint the expression of IFN-β and downstream ISG56 (177). Kaposi’s sarcoma-associated herpesvirus (KSHV) ORF64 is an envelope protein with DUB activity. ORF64 plays a crucial role in promoting KSHV replication and antagonizing RIG-I-mediated innate immunity. This is mainly because ORF64 can target RIG-I de-ubiquitination, inhibiting its downstream signal transduction (178, 179).

Viruses can also promote their own replication by encoding E3 or proteins that mimic E3 functions. For example, the ORF73 protein encoded by the murine herpes virus contains a unique SOCS box-like motif that can mediate the assembly of the ElonginC/Cullin5/SOCS-like complex, and the complex mimics the function of the E3 ligase to catalyze the ubiquitination and subsequent proteasome degradation of p65/RelA, ultimately blocking the activation of NF-κB and promoting the continued infection of the virus (180). Herpes Simplex Virus Type 1 (HSV-1) infected cell protein 0 (ICP0) protein is a virus-encoded E3. It was found that ICP0 interacts with NF-κB subunits p65 and p50, and mediates ubiquitination and proteasome degradation of p50 through its RF domain, thereby restricting the expression of NF-κB dependent genes and achieving immune evasion (181). Toscana virus (TOSV) is an intravenous virus, belonging to the Bunyaco virus. It was found that TOSV NSs protein binds to RIG-I and targets its degradation in a proteasome manner to interrupt the production of IFN-β. Further research found that TOSV NSs protein has E3 activity, mainly through its CTD domain and amino-terminal to exert enzyme activity (182, 183). Besides, HSV-2 ICP22 also limits IFN-I signal transduction. The specific mechanism is that ICP22 promotes the ubiquitination of STAT1, STAT2, and IRF9, and ICP22 plays the role of E3s to catalyze the ubiquitination and degradation of IFN-stimulated gene factor 3.

Fish viruses also utilize the ubiquitin system to escape immune mechanisms. For example, during spring viremia of carp virus (SVCV) infection, the viral N protein inhibits the K63-linked ubiquitination of p53 and assists its degradation. On the contrary, SVCV P protein stabilizes p53 by binding to p53 and promoting its K63-linked ubiquitination. Therefore, the fish virus SVCV uses this unique way to antagonize p53-mediated innate immunity (184) (**Table 3**).

**Table 3:** Express virus-encoded E3s/DUBs.

Virus protein	Target	Effect
Herpes virus ORF73	p65/RelA	NF-κB signal ↓
HSV-1 ICP0	p65 and p50	NF-κB signal ↓
TOSV NSs	RIG-I	IFN-I production ↓
HSV-2 ICP22	STAT1, STAT2 and IRF9	IFN-I production ↓
SVCV N	p53	p53 mediated innate immunity ↓
Arterial virus PLP2	RIG-I	RLR signal ↓
Nairo virus OTU	RIG-I	RLR signal ↓
Hepatitis virus A59	TBK1, IRF3	IFN-I production ↓
NSP3		
FMDV pro	RIG-I, TRAF3, TRAF6 and TBK1	IFN-I production ↓
Human coronavirus and SARS-CoV PLP	STING, RIG-I, TBK1 and IRF-3	IFN-I production ↓
PEDV PLP2	RIG-I and STING	IFN-I production ↓
HEV ORF1	RIG-I and TBK-1	RLR signal ↓
HSV VP1-2	STING	IFN-I production ↓
HSV UL36USP	IκB-α	NF-κB signal ↓, IFN-I production ↓
HBV X	RIG-I and TRAF3	Innate immunity ↓
SVV 3Cpro	RIG-I, TBK1, and TRAF3	Expression of IFN-β and ISG56 ↓
KSHV ORF64	RIG-I	RLR signal ↓

↑ increases; ↓ decreases.

### Hijack the host’s E3s/DUBs

As intracellular parasites, the viruses exploit various methods to manipulate the E3s/DUBs encoded by the host to avoid innate immunity. For example, during severe fever with thrombocytopenia syndrome virus infection, the virus utilizes its self-encoded NSs protein to hijack TRIM25 into viral inclusion bodies to inhibit TRIM25-catalyzed RIG-I K63-linked ubiquitination, thereby promoting early host proliferation (185). The V protein of Newcastle disease virus (NDV) can interact with MAVS, and the V protein can recruit E3 RNF5 upon NDV infection. RNF5 further promotes the ubiquitination and proteasomal degradation of MAVS, thereby inhibiting the production of IFN-I and assisting the proliferation of the virus (186). During DENV infection, the E3 seven in absentia homolog 1 (SIAH1) is activated by an unfolded protein response. Further studies have shown that SIAH1 binds to MyD88 and mediates its ubiquitination and proteasome degradation, thereby inhibiting the innate immune response and promoting virus replication (187).

The specific mechanism by which HBV infection cannot cause IFN-I antiviral signals is unknown. A recent study found that HBV infection induces parkin expression. Parkin can bind to MAVS and recruit LUBAC to mitochondria, thereby accumulating unanchored linear ubiquitin chains on MAVS, which damages the MAVS signaling body and weakens the activation of IRF3, eventually blocking IFN-I signal induction during HBV infection (188). Fish viruses can also use host-encoded E3s. Red-spotted grouper nervous necrosis virus (RGNNV) infection can cause the E3 LjRNF114 of sea perch (Lateolabrax japonicus) to up-regulate, and the up-regulated LjRNF114 boosts the binding of MAVS and TRAF3 to the ubiquitin chains linked to K27 and K48, which leads to MAVS and TRAF3 degrade, RLR signal transduction is destroyed, and ultimately facilitate virus infection (189).

Some viruses can also hijack the host’s DUBs by manipulating the microRNA to facilitate their replication. For example, during DENV infection, NS1 induces cells to release lots of external vesicles containing miR-148a, while miR-148a further inhibits the expression of USP33 protein, and USP33 affects the stability of activating transcription factor 3 (ATF3) protein by deubiquitinating it. It is worth noting that ATF3 is an essential inhibitor of pro-inflammatory gene expressions such as TNF-α, and NF-κB. Hence DENV facilitates the inflammatory response of the central nervous system through this pathway (190). Similarly, enterovirus 71 3Cpro inhibits the expression of host miR-526, and miR-526 targets the DUB CYLD. Overexpression of 3Cpro will cause the down-regulation of miR-526 but facilitate the up-regulation of CYLD. CLYD further eliminates the K63-linked ubiquitin chains of RIG-I, thereby blocking RIG-I-mediated immune signals and promoting virus replication (119, 191).

DUB USP27X is an antiviral signaling inhibitor. Studies have found that USP27X interacts with RIG-I CARD and removes the K63-linked polyubiquitin chains of RIG-I, thereby negatively regulating RIG-I mediated antiviral signal (192). Viral infection can cause upregulation of OTUD1, and OTUD1 as a DUB can attenuate the K6-linked ubiquitination of IRF3 to affect the DNA-binding ability of IRF3, thereby antagonizing the IRF3-mediated innate immune pathway (193). Bovine viral diarrhea virus infection of Madin-Darby bovine kidney cells will induce strong expression of DNA damage-inducible transcript 3 (DDIT3) protein and mRNA, and overexpression of DDIT3 will assist viral replication. The specific mechanism is: DDIT3 expression induces the production of OTUD1, which promotes the upregulation of Smurf1 expression by deubiquitinating Smurf1, and Smurf1 as E3 will degrade MAVS in a ubiquitin-dependent manner to inhibit IFN-I signaling (194). Studies have found that UBE2S binds to TBK1 and recruits DUB USP15 to specifically remove the K63-linked polyubiquitin chains on TBK1, thereby antagonizing the expression of IFN-I. In addition, the deletion of UBE2S suppresses virus replication and boosts the antiviral response in cells, which also confirms the above conclusions (195). Similarly, RNA viruses can also target the ubiquitination and proteasome degradation of IRF3 by hijacking the host’s E2 UBE2J1, thereby restraining the induction of IFN-I and promoting virus replication (**Table 4**) (196).

**Table 4:** Hijack the host's E3s/DUBs.

Virus protein	Target	Effect
SFTSV NSs	TRIM25	Viral infection ↑
NDV V	RNF5	IFN-I production ↓
DENV	SIAH1	MyD88 mediated innate immunity ↓
HBV	Parkin	IFN-I production ↓
RGNNV	LjRNF114	RLR signal ↓
DENV	USP33	Inflammatory response ↑
Enterovirus 71 3Cpro	CYLD	RLR signal ↓
BVDV	OTUD1	IFN-I production ↓

↑ increases; ↓ decreases.

## Conclusion and future perspective

Numerous lines of evidence support the important regulatory role of the ubiquitin system in host-pathogen interactions. Although significant progress has been made in exploring the function and mechanism of the ubiquitin system in regulating innate immunity in the past few decades, there are still many research gaps. Firstly, we need to dissect the underlying molecular details: which protein targets, E3s or DUBs, are responsible for the observed phenotype? How are these E3s and DUBs regulated under normal and infectious conditions? Considering that a particular protein is usually regulated by several different E3s and/or DUBs, attention should be paid to clarifying whether these different enzymes have redundant functions or whether they perform related functions in a cell-type and environment-related manner. These studies will further reveal the crosstalk between immune signal cascades and reveal a functional and self-regulated whole. Secondly, in order to understand the specific mechanism of different forms of PTMs of innate signaling molecules observed in different organelles and responding to different pathogenic stimuli, we need to develop and apply new “omics” to comprehensively and dynamically understand the innate immune system.

Finally, many gaps remain in our understanding of pathogen immune evasion strategies associated with the ubiquitin system. How are these pathogen-derived E3s and DUBs regulated upon entry into the host environment? How do these multifunctional effector proteins coordinate their various activities under physiological conditions? Importantly, these explorations will provide a theoretical basis for the development of vaccine strains to curb infectious diseases and effective treatment methods in the future.

## Author contributions

SH conducted literature collection and completed the manuscript writing. RJ improved the manuscript and coordinated the project. AC, MW, ZY, JH revised the manuscript. All authors have read and agreed to the published version of the manuscript.

## Funding

This work was supported by the National Natural Science Foundation of China (32172833), Natural Science Foundation of Sichuan Province (2022NSFSC0078), the earmarked fund for China Agriculture Research System of MOF and MARA, and Sichuan Veterinary Medicine and Drug Innovation Group of China Agricultural Research System (SCCXTD-2021-18).

## Conflict of interest

The authors declare that the research was conducted in the absence of any commercial or financial relationships that could be construed as a potential conflict of interest.

## Publisher’s note

All claims expressed in this article are solely those of the authors and do not necessarily represent those of their affiliated organizations, or those of the publisher, the editors and the reviewers. Any product that may be evaluated in this article, or claim that may be made by its manufacturer, is not guaranteed or endorsed by the publisher.
